# [2,6-Bis(di-*tert*-butyl­phosphinometh­yl)­phen­yl-κ^3^ *P*,*C* ^1^,*P*′](nitrato-κ*O*)nickel(II)

**DOI:** 10.1107/S1600536808032376

**Published:** 2008-10-15

**Authors:** Brian J. Boro, Diane A. Dickie, Eileen N. Duesler, Karen I. Goldberg, Richard A. Kemp

**Affiliations:** a Department of Chemistry and Chemical Biology, MSC03 2060, 1 University of New Mexico, Albuquerque, NM 87131, USA; b Department of Chemistry, University of Washington, Seattle, WA 98195, USA; c Advanced Materials Laboratory, Sandia National Laboratories, 1001 University Boulevard SE, Albuquerque, NM 87106, USA

## Abstract

The Ni^II^ atom in the title compound, [Ni(C_24_H_43_P_2_)(NO_3_)], adopts a distorted square-planar geometry with the P atoms in a *trans* arrangement. The compound contains a twofold rotational axis with the nitrate group offset from this axis, except for an O atom of the nitrate group, generating two positions of 50% occupancy for the other atoms of the nitrate group.

## Related literature

The synthetic preparation was adopted from that employed to prepare the Pd analogue (Cámpora *et al.*, 2004 ▶). For the crystallographic characterization of the Pd analogue, see: Olsson *et al.* (2007 ▶). For the crystallographic characterization of the starting {2,6-bis­[(di-*tert*-butyl­phosphino)meth­yl]phenyl}chloridonickel complex, see: Boro *et al.* (2008 ▶). For related literature, see: Denney *et al.* (2006 ▶); Johansson *et al.* (2007 ▶); Keith *et al.* (2006 ▶).
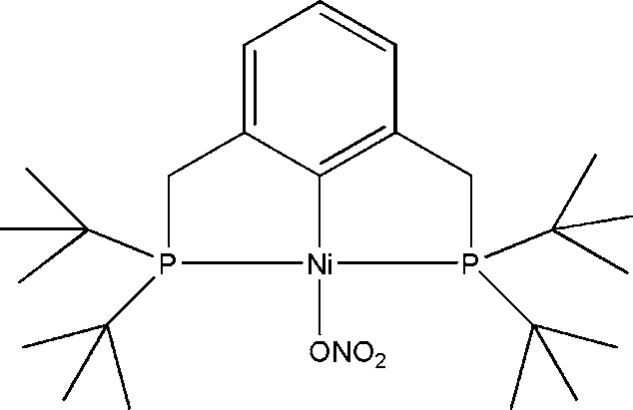


## Experimental

### 

#### Crystal data

[Ni(C_24_H_43_P_2_)(NO_3_)]
*M* *_r_* = 514.24
Orthorhombic, 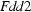

*a* = 24.0023 (14) Å
*b* = 12.6350 (6) Å
*c* = 17.6528 (6) Å
*V* = 5353.5 (4) Å^3^
*Z* = 8
Mo *K*α radiation
μ = 0.87 mm^−1^
*T* = 225 (2) K
0.50 × 0.40 × 0.20 mm

#### Data collection

Bruker X8 APEXII CCD area-detector diffractometer
Absorption correction: multi-scan (*SADABS*; Bruker, 2004 ▶) *T*_min_ = 0.671, *T*_max_ = 0.846
33484 measured reflections
4078 independent reflections
3703 reflections with *I* > 2σ(*I*)
*R*_int_ = 0.037

#### Refinement

*R*[*F* ^2^ > 2σ(*F* ^2^)] = 0.030
*wR*(*F* ^2^) = 0.099
*S* = 0.81
4078 reflections
156 parameters
1 restraint
H-atom parameters constrained
Δρ_max_ = 0.38 e Å^−3^
Δρ_min_ = −0.38 e Å^−3^
Absolute structure: Flack (1983 ▶), 1976 Freidel pairs
Flack parameter: 0.012 (10)

### 

Data collection: *APEX2* (Bruker, 2007 ▶); cell refinement: *APEX2*; data reduction: *APEX2*; program(s) used to solve structure: *SHELXS97* (Sheldrick, 2008 ▶); program(s) used to refine structure: *SHELXL97* (Sheldrick, 2008 ▶); molecular graphics: *DIAMOND* (Brandenburg, 1999 ▶); software used to prepare material for publication: *publCIF* (Westrip, 2008 ▶).

## Supplementary Material

Crystal structure: contains datablocks I, global. DOI: 10.1107/S1600536808032376/at2643sup1.cif

Structure factors: contains datablocks I. DOI: 10.1107/S1600536808032376/at2643Isup2.hkl

Additional supplementary materials:  crystallographic information; 3D view; checkCIF report

## Figures and Tables

**Fig. 1.**
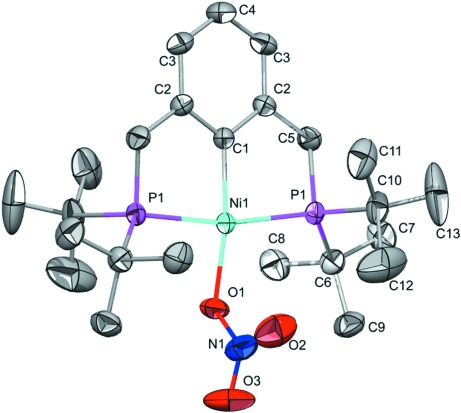
View of the title compound showing full numbering scheme. Ellipsoids are shown at 50% probability and hydrogen atoms have been removed for clarity. Only one of the two symmetry equivalent positions for the nitrate group is shown.

**Fig. 2.**
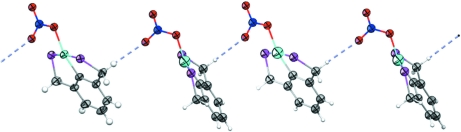
Hydrogen bonding in (I). For clarity the tert-butyl substituents on P have been removed.

## supplementary crystallographic information

### Comment

The title compound, (I) (Fig. 1), was prepared from {2,6-bis[(*di*-*tert*-butylphosphino)methyl]phenyl}chloridonickel(II) (Boro *et al.*, 2008) *via* a synthesis adopted from the preparation of the Pd analogue (Cámpora *et al.*, 2004). Our research has been directed towards the activation of molecular oxygen using pincer complexes of the late transition metals (Denney *et al.*, 2006; Keith *et al.*, 2006). Compound (I) was prepared as a precursor along the path of the attempted synthesis of a Ni^II^-hydroperoxide.

The compound is bisected by a twofold rotational axis running through C4, C1, Ni1, and O3. The nitrate group is offset from this axis with a C1—Ni1—O1 angle of 164.64 (11)°. As a result of this symmetry induced disorder the nitrate group occupies two positions each with 50% occupancy. The Ni1—O1 bond length 1.976 (3) Å is significantly shorter than the corresponding Pd—O bond length 2.164 (2) Å in the Pd analogue (Johansson *et al.* 2007). This not surprising, given the smaller size of the Ni atom. The M—C and M—P bonds were also shorter in the Ni compound compared to the Pd. The P—M—P angle, however was closer to the ideal linear geometry with Ni (168.87 (3)°) than Pd (163.41 (3)°).

In their report on the Pd analogue of (I), Johansson *et al.* (2007) observed non-traditional hydrogen bonding between one of the nitrato O atoms and a hydrogen from the methylene arm of the pincer ligand to form a zigzag chain. The C—H···O interaction measured 3.263 (4) Å (C···O) with an angle of 137°. The same pattern is observed in I (Fig. 2) between C5—H5b···O2, with corresponding measurements of 3.5616 (54) Å and 171.81 (18)°.

### Experimental

{2,6-Bis[(*di*-*tert*-butylphosphino)methyl]phenyl}chloronickel(II) (0.135 g, 0.28 mmol) and AgNO_3_ (0.05 g, 0.29 mmol) were combined with THF (20 ml) and the solution was stirred at room temperature for 48 h. The THF was then removed under vacuum and the product was extracted into diethyl ether (15 ml). After filtering, the solution was layered with hexanes and placed in a 238 K freezer. The product crystallized as orange-brown prisms.

Yield 0.118 g, 82%. ^1^H NMR (250 MHz, C_6_D_6_): 6.93 (d, J_HH_ = 7.5 Hz, 1H, Ar-*H_para_*), 6.68 (t, J_HH_ = 7.5 Hz, 2H, Ar-*H_meta_*), 2.80 (virtual t, 4H, J_HP_ = 7.15 Hz,*CH*_2_), 1.25 (virtual t, 36H, J = 13.1 Hz C(C*H*_3_)_3_) p.p.m.. ^13^C{^1^H} (63 MHz, C_6_D_6_): 152.8 (t, ^2^J_PC_ = 11.6 Hz, Ar-*C_ipso_*), 147.1 (virtual t, J_CP_= 32.3 Hz, Ar-*C_ortho_*), 125.8 (s, Ar-*C_para_*), 122.1 (virtual t, J_CP_ = 15.8 Hz, Ar-*C_para_*), 34.5 (virtual t, J_CP_ = 13.2 Hz, P*C*H_2_), 32.7 (virtual t, J_CP_ = 24.6 Hz, P*C*(CH_3_)_3_), 29.6 (s, *C*H_3_) p.p.m.. ^31^P{^1^H} (101 MHz, C_6_D_6_): 69.5 (*s*) p.p.m..

### Refinement

Hydrogen atoms were included at geometrically idealized positions with C—H distances at the range of 0.93 - 0.98 Å and were treated as riding on their respective heavy atoms. The isotropic thermal parameters of the hydrogen atoms were fixed at 1.2 or 1.5 *U*_eq_ of the parent atom.

### Crystal data

**Table:**

[Ni(C_24_H_43_P_2_)(NO_3_)]	*F*(000) = 2208
*M**_r_* = 514.24	*D*_x_ = 1.276 Mg m^−^^3^
Orthorhombic, *F**d**d*2	Mo *K*α radiation, λ = 0.71073 Å
Hall symbol: F 2 -2d	Cell parameters from 9535 reflections
*a* = 24.0023 (14) Å	θ = 2.2–30.3°
*b* = 12.6350 (6) Å	µ = 0.87 mm^−^^1^
*c* = 17.6528 (6) Å	*T* = 225 K
*V* = 5353.5 (4) Å^3^	Cut-prism, orange–brown
*Z* = 8	0.50 × 0.40 × 0.20 mm

### Data collection

**Table:**

Bruker SMART CCD area-detector diffractometer	4078 independent reflections
Radiation source: fine-focus sealed tube	3703 reflections with *I* > 2σ(*I*)
graphite	*R*_int_ = 0.037
φ and ω scans	θ_max_ = 30.5°, θ_min_ = 2.9°
Absorption correction: multi-scan (SADABS; Bruker, 2004)	*h* = −34→34
*T*_min_ = 0.671, *T*_max_ = 0.846	*k* = −18→18
33484 measured reflections	*l* = −25→25

### Refinement

**Table:**

Refinement on *F*^2^	Secondary atom site location: difference Fourier map
Least-squares matrix: full	Hydrogen site location: inferred from neighbouring sites
*R*[*F*^2^ > 2σ(*F*^2^)] = 0.030	H-atom parameters constrained
*wR*(*F*^2^) = 0.099	*w* = 1/[σ^2^(*F*_o_^2^) + (0.1*P*)^2^ + 0.25*P*] where *P* = (*F*_o_^2^ + 2*F*_c_^2^)/3
*S* = 0.81	(Δ/σ)_max_ = 0.001
4078 reflections	Δρ_max_ = 0.38 e Å^−^^3^
156 parameters	Δρ_min_ = −0.38 e Å^−^^3^
1 restraint	Absolute structure: Flack (1983), 1976 Freidel pairs
Primary atom site location: structure-invariant direct methods	Flack parameter: 0.012 (10)

### Special details

**Table:**

Geometry. All e.s.d.'s (except the e.s.d. in the dihedral angle between two l.s. planes) are estimated using the full covariance matrix. The cell e.s.d.'s are taken into account individually in the estimation of e.s.d.'s in distances, angles and torsion angles; correlations between e.s.d.'s in cell parameters are only used when they are defined by crystal symmetry. An approximate (isotropic) treatment of cell e.s.d.'s is used for estimating e.s.d.'s involving l.s. planes.
Refinement. Refinement of *F*^2^ against ALL reflections. The weighted *R*-factor *wR* and goodness of fit *S* are based on *F*^2^, conventional *R*-factors *R* are based on *F*, with *F* set to zero for negative *F*^2^. The threshold expression of *F*^2^ > σ(*F*^2^) is used only for calculating *R*-factors(gt) *etc*. and is not relevant to the choice of reflections for refinement. *R*-factors based on *F*^2^ are statistically about twice as large as those based on *F*, and *R*- factors based on ALL data will be even larger.

### Fractional atomic coordinates and isotropic or equivalent isotropic displacement parameters (Å^2^)

**Table:**

	*x*	*y*	*z*	*U*_iso_*/*U*_eq_	Occ. (<1)
Ni1	0.2500	0.2500	0.594610 (14)	0.02536 (9)	
P1	0.232841 (19)	0.07823 (3)	0.58242 (3)	0.02639 (10)	
C1	0.2500	0.2500	0.48634 (16)	0.0263 (5)	
C2	0.22870 (9)	0.16438 (14)	0.44415 (10)	0.0293 (3)	
C3	0.22918 (11)	0.16401 (17)	0.36544 (11)	0.0381 (4)	
H3	0.2153	0.1051	0.3390	0.046*	
C4	0.2500	0.2500	0.32575 (19)	0.0443 (8)	
H4	0.2500	0.2500	0.2725	0.053*	
C5	0.20438 (10)	0.07210 (16)	0.48626 (12)	0.0357 (4)	
H5A	0.2149	0.0054	0.4618	0.043*	
H5B	0.1636	0.0770	0.4875	0.043*	
C6	0.17578 (10)	0.02125 (17)	0.64252 (13)	0.0356 (4)	
C7	0.15103 (12)	−0.08133 (18)	0.60934 (17)	0.0492 (6)	
H7A	0.1803	−0.1337	0.6038	0.074*	
H7B	0.1348	−0.0665	0.5602	0.074*	
H7C	0.1225	−0.1082	0.6431	0.074*	
C8	0.13040 (11)	0.1061 (2)	0.64349 (17)	0.0490 (5)	
H8A	0.1001	0.0833	0.6759	0.073*	
H8B	0.1166	0.1170	0.5925	0.073*	
H8C	0.1458	0.1718	0.6627	0.073*	
C9	0.19464 (14)	0.00157 (19)	0.72359 (15)	0.0484 (6)	
H9A	0.1623	−0.0057	0.7561	0.073*	
H9B	0.2171	0.0607	0.7408	0.073*	
H9C	0.2166	−0.0628	0.7256	0.073*	
C10	0.29710 (10)	−0.00713 (16)	0.58017 (16)	0.0434 (5)	
C11	0.33545 (12)	0.0368 (3)	0.5189 (2)	0.0635 (9)	
H11A	0.3615	−0.0176	0.5032	0.095*	
H11B	0.3558	0.0970	0.5388	0.095*	
H11C	0.3134	0.0590	0.4757	0.095*	
C12	0.32896 (19)	0.0019 (4)	0.6540 (3)	0.100 (2)	
H12A	0.3293	−0.0663	0.6793	0.150*	
H12B	0.3110	0.0538	0.6864	0.150*	
H12C	0.3669	0.0240	0.6437	0.150*	
C13	0.28517 (17)	−0.1214 (3)	0.5629 (4)	0.106 (2)	
H13A	0.2705	−0.1274	0.5118	0.159*	
H13B	0.2580	−0.1484	0.5986	0.159*	
H13C	0.3193	−0.1621	0.5670	0.159*	
N1	0.2619 (2)	0.2312 (5)	0.7546 (2)	0.0478 (13)	0.50
O1	0.22947 (16)	0.2640 (3)	0.70255 (19)	0.0394 (7)	0.50
O2	0.3061 (2)	0.1884 (5)	0.7355 (3)	0.0732 (13)	0.50
O3	0.2500	0.2500	0.8206 (2)	0.0869 (14)	

### Atomic displacement parameters (Å^2^)

**Table:**

	*U*^11^	*U*^22^	*U*^33^	*U*^12^	*U*^13^	*U*^23^
Ni1	0.03377 (16)	0.01934 (13)	0.02296 (14)	−0.00129 (11)	0.000	0.000
P1	0.0305 (2)	0.01967 (19)	0.0290 (2)	0.00015 (15)	0.00072 (17)	0.00082 (15)
C1	0.0312 (13)	0.0241 (12)	0.0238 (11)	0.0033 (8)	0.000	0.000
C2	0.0344 (8)	0.0280 (8)	0.0256 (8)	0.0006 (7)	−0.0019 (7)	−0.0025 (6)
C3	0.0478 (11)	0.0397 (11)	0.0267 (9)	−0.0013 (8)	−0.0025 (8)	−0.0070 (7)
C4	0.059 (2)	0.0497 (19)	0.0237 (12)	0.0046 (13)	0.000	0.000
C5	0.0468 (11)	0.0289 (9)	0.0314 (9)	−0.0077 (8)	−0.0027 (8)	−0.0030 (7)
C6	0.0378 (10)	0.0323 (8)	0.0368 (10)	−0.0059 (8)	0.0047 (8)	0.0017 (7)
C7	0.0514 (13)	0.0399 (11)	0.0564 (14)	−0.0172 (10)	0.0099 (11)	−0.0066 (9)
C8	0.0387 (11)	0.0530 (13)	0.0552 (14)	0.0026 (10)	0.0096 (10)	−0.0039 (11)
C9	0.0621 (16)	0.0468 (14)	0.0364 (11)	−0.0120 (11)	0.0025 (10)	0.0099 (9)
C10	0.0380 (10)	0.0317 (10)	0.0606 (15)	0.0095 (7)	0.0119 (10)	0.0072 (9)
C11	0.0397 (12)	0.0590 (16)	0.092 (2)	0.0147 (12)	0.0217 (15)	0.0184 (17)
C12	0.061 (2)	0.168 (6)	0.070 (3)	0.056 (3)	−0.0012 (18)	0.028 (2)
C13	0.069 (2)	0.0339 (14)	0.215 (7)	0.0129 (13)	0.034 (3)	−0.011 (2)
N1	0.057 (4)	0.052 (4)	0.0350 (19)	−0.016 (2)	−0.0114 (18)	0.0079 (18)
O1	0.0488 (17)	0.0462 (17)	0.0233 (14)	−0.0022 (15)	0.0012 (13)	−0.0048 (12)
O2	0.054 (2)	0.104 (4)	0.062 (3)	0.012 (3)	−0.011 (2)	0.019 (3)
O3	0.098 (3)	0.137 (4)	0.0265 (13)	−0.012 (2)	0.000	0.000

### Geometric parameters (Å, °)

**Table:**

Ni1—C1	1.911 (3)	C7—H7C	0.9700
Ni1—O1^i^	1.976 (3)	C8—H8A	0.9700
Ni1—O1	1.976 (3)	C8—H8B	0.9700
Ni1—P1^i^	2.2195 (4)	C8—H8C	0.9700
Ni1—P1	2.2195 (4)	C9—H9A	0.9700
P1—C5	1.831 (2)	C9—H9B	0.9700
P1—C6	1.876 (2)	C9—H9C	0.9700
P1—C10	1.882 (2)	C10—C13	1.503 (4)
C1—C2^i^	1.409 (2)	C10—C12	1.516 (6)
C1—C2	1.409 (2)	C10—C11	1.525 (4)
C2—C3	1.389 (3)	C11—H11A	0.9700
C2—C5	1.501 (3)	C11—H11B	0.9700
C3—C4	1.386 (3)	C11—H11C	0.9700
C3—H3	0.9400	C12—H12A	0.9700
C4—C3^i^	1.386 (3)	C12—H12B	0.9700
C4—H4	0.9400	C12—H12C	0.9700
C5—H5A	0.9800	C13—H13A	0.9700
C5—H5B	0.9800	C13—H13B	0.9700
C6—C9	1.521 (3)	C13—H13C	0.9700
C6—C8	1.528 (4)	N1—O3	1.223 (5)
C6—C7	1.541 (3)	N1—O2	1.239 (7)
C7—H7A	0.9700	N1—O1	1.273 (5)
C7—H7B	0.9700	O3—N1^i^	1.223 (5)
			
C1—Ni1—O1^i^	164.64 (11)	C6—C8—H8B	109.5
C1—Ni1—O1	164.64 (11)	H8A—C8—H8B	109.5
C1—Ni1—P1^i^	84.437 (14)	C6—C8—H8C	109.5
O1^i^—Ni1—P1^i^	97.74 (10)	H8A—C8—H8C	109.5
O1—Ni1—P1^i^	93.00 (10)	H8B—C8—H8C	109.5
C1—Ni1—P1	84.435 (14)	C6—C9—H9A	109.5
O1^i^—Ni1—P1	93.00 (10)	C6—C9—H9B	109.5
O1—Ni1—P1	97.74 (10)	H9A—C9—H9B	109.5
P1^i^—Ni1—P1	168.87 (3)	C6—C9—H9C	109.5
C5—P1—C6	103.63 (10)	H9A—C9—H9C	109.5
C5—P1—C10	105.18 (12)	H9B—C9—H9C	109.5
C6—P1—C10	112.97 (10)	C13—C10—C12	110.1 (3)
C5—P1—Ni1	101.56 (7)	C13—C10—C11	108.7 (3)
C6—P1—Ni1	117.12 (7)	C12—C10—C11	106.1 (3)
C10—P1—Ni1	114.22 (8)	C13—C10—P1	113.5 (2)
C2^i^—C1—C2	116.2 (3)	C12—C10—P1	110.6 (2)
C2^i^—C1—Ni1	121.90 (13)	C11—C10—P1	107.52 (16)
C2—C1—Ni1	121.90 (13)	C10—C11—H11A	109.5
C3—C2—C1	121.9 (2)	C10—C11—H11B	109.5
C3—C2—C5	119.73 (18)	H11A—C11—H11B	109.5
C1—C2—C5	118.39 (19)	C10—C11—H11C	109.5
C4—C3—C2	120.4 (2)	H11A—C11—H11C	109.5
C4—C3—H3	119.8	H11B—C11—H11C	109.5
C2—C3—H3	119.8	C10—C12—H12A	109.5
C3^i^—C4—C3	119.3 (3)	C10—C12—H12B	109.5
C3^i^—C4—H4	120.4	H12A—C12—H12B	109.5
C3—C4—H4	120.4	C10—C12—H12C	109.5
C2—C5—P1	106.33 (13)	H12A—C12—H12C	109.5
C2—C5—H5A	110.5	H12B—C12—H12C	109.5
P1—C5—H5A	110.5	C10—C13—H13A	109.5
C2—C5—H5B	110.5	C10—C13—H13B	109.5
P1—C5—H5B	110.5	H13A—C13—H13B	109.5
H5A—C5—H5B	108.7	C10—C13—H13C	109.5
C9—C6—C8	108.4 (2)	H13A—C13—H13C	109.5
C9—C6—C7	109.6 (2)	H13B—C13—H13C	109.5
C8—C6—C7	108.6 (2)	O1^i^—N1—O3	165.1 (6)
C9—C6—P1	112.18 (18)	O1^i^—N1—O2	64.8 (4)
C8—C6—P1	104.93 (15)	O3—N1—O2	123.0 (4)
C7—C6—P1	112.90 (16)	O3—N1—O1	118.8 (4)
C6—C7—H7A	109.5	O2—N1—O1	117.9 (5)
C6—C7—H7B	109.5	N1^i^—O1—O2^i^	69.6 (4)
H7A—C7—H7B	109.5	O2^i^—O1—N1	104.4 (4)
C6—C7—H7C	109.5	N1^i^—O1—Ni1	152.8 (5)
H7A—C7—H7C	109.5	O2^i^—O1—Ni1	133.8 (4)
H7B—C7—H7C	109.5	N1—O1—Ni1	121.0 (3)
C6—C8—H8A	109.5		

Symmetry codes: (i) −*x*+1/2, −*y*+1/2, *z*.
